# Effects of oral contraceptives on the quality of life of women with polycystic ovary syndrome: a crossover randomized controlled trial

**DOI:** 10.1186/s12955-020-01544-4

**Published:** 2020-08-31

**Authors:** Mina Amiri, Fatemeh Nahidi, Razieh Bidhendi Yarandi, Davood Khalili, Maryam Tohidi, Fahimeh Ramezani Tehrani

**Affiliations:** 1 Reproductive Endocrinology Research Center, Research Institute for Endocrine Sciences, Shahid Beheshti University of Medical Sciences, No 24, Parvane Street, Yaman Street, Velenjak, Tehran, Iran; 2 Department of Midwifery and Reproductive Health, Faculty of Nursing and Midwifery, Shahid Beheshti University of Medical Sciences, Tehran, Iran; 3 Department of Epidemiology and biostatistics, School of Public Health, Tehran University of Medical Sciences, Tehran, Iran; 4 Prevention of Metabolic Disorders Research Center, Research Institute for Endocrine Sciences, Shahid Beheshti University, Tehran, Iran

**Keywords:** Polycystic ovary syndrome (PCOS), Oral contraceptive (OC), Quality of life (QOL)

## Abstract

**Background and objective:**

A limited number of studies have evaluated the effects of oral contraceptives (OCs) on the quality of life (QOL) of polycystic ovary syndrome (PCOS) patients. This study aimed to compare the effects of using OCs containing levonorgestrel (LNG) and those containing desogestrel (DSG), cyproterone acetate (CPA) or drospirenone (DRSP) for 6 months on the QOL with PCOS.

**Methods:**

In this crossover randomized controlled 6-arm trial, 200 eligible patients with PCOS scheduled for OC therapy were randomly assigned to one of the 6 study arms. All 6 arms include two 6-month treatment periods, one period with OCs containing LNG, and the other with each of the 3 OCs containing DSG, CPA, or DRSP. Outcomes of interest were the total score of QOL and its domains, which were assessed using a specific and valid health-related quality of life questionnaire for PCOS, which is consisted of six domains, including psychosocial–emotional, self-image, fertility, sexual function, hirsutism, and obesity– menstrual disorders.

**Results:**

Finally, a total of 88 patients were analyzed for this study. The results showed that use of OCs containing DSG, CPA, and DRSP for 3 months was not associated with significant differences in the total scores of QOL compared to those OCs containing LNG, whereas, after 6 months of treatment, patients treated with OCs containing CPA had more improvements in their total scores of QOL, in comparison to OCs containing LNG (*P* < 0.042). We found no significant differences in QoL domains, including psychosocial–emotional, self-image, fertility, sexual function, hirsutism, and obesity-menstrual disorders after 3–6 months of treatment with DSG, CPA, or DRSP, compared to LNG. The sequence and period effects were not significant in any of the analyses at 3 and 6 months of treatment. The carry-over effect was not significant for most outcomes assessed.

**Conclusions:**

This crossover study demonstrated non-inferiority of OCs with newer generation progestins on different domains of QOL, in comparison with older compounds, although usage of products containing CPA was significantly associated with more improvement in total QOL of PCOS patients, compared to those containing LNG after 6-month of treatment.

**Trial registration:**

IRCT201702071281N2.

## Background

Polycystic ovary syndrome (PCOS), the most common endocrinopathy in females, is characterized by hyperandrogenism, oligo-anovulation, and polycystic ovaries leading to hirsutism, acne, hair loss, menstrual irregularity, and infertility [1]. This syndrome is also associated with an increased risk of metabolic disturbances, including obesity, hyperinsulinemia, insulin resistance, and dyslipidemia, which predispose women to cardiovascular diseases and diabetes mellitus [2].

In addition to endocrine and metabolic disorders, women with PCOS frequently experience several psychological comorbidities, such as depression, anxiety, sexual dysfunction, and social problems [3–5], which can negatively influence their feminine identity and health-related quality of life (QOL) [4, 6]. Indeed, the QOL of women with PCOS is mainly affected by the clinical features of this syndrome and also depressive symptoms, anxiety, poor body image and low self-esteem [7].

There are several options for PCOS treatment, which individualize depending on clinical manifestations, needs, and preferences of each patient. The goals of all therapies are to improve biochemical, and clinical outcomes of PCOS as well as the QOL. It is well-documented that oral contraceptives (OCs), regardless of their progestin component, are considered as first-line treatment for women with PCOS, they can decrease menstrual irregularity and hyperandrogenism, and leads to improvements in specific domains of QOL in patients with PCOS [6]. In some countries, such as Iran, OCs containing a second-generation progestin, levonorgestrel (LNG), are still frequently used as affordable and cost effective remedies in both healthy women and patients with irregular menstrual or HA symptoms and can be as effective as those OCs with antiandrogenic properties [8].

Despite numerous studies on the association between PCOS and QOL [6, 7, 9–11], a limited number of studies have evaluated the effects of OCs on the QOL of PCOS patients [12–15]. Besides, to the best our knowledge, there is no study comparing the effectiveness of these pharmacological treatments on the QOL of patients based on their progestin component. Hence, the aim of this crossover randomized controlled trial was to compare the effects of using OCs containing levonorgestrel (LNG) and those containing desogestrel (DSG), cyproterone acetate (CPA) or drospirenone (DRSP) on the QOL of patients with PCOS, over a period of 6 months.

## Methods

### Study design, ethics approval and participants

This study is a crossover randomized controlled 6-arm trial designed based on the requirements of the Consolidated Standards of Reporting Trials (CONSORT) [16] and initiated in February 2016. This clinical trial is registered in the Iran Registry of Clinical Trials (Registration number: IRCT201702071281N2). Ethics approval was obtained from the ethics committee of Shahid Beheshti University of Medical Sciences, Tehran, Iran. (Ethics Code: IR.SBMU.ENDOCRINE.REC.1396.425). We obtained written informed consent from all eligible participants, following study content being clearly explained to the subjects by the trial assistant; all research tools, including questionnaires, were completely anonymous.

Patients with PCOS diagnosed based on the Androgen Excess Society (AES) criteria (age 18–45 years) were recruited at the endocrine out-patients clinic of the Research Institute for Endocrine Sciences (RIES) of the Shahid Beheshti University of Medical Sciences, Tehran, Iran. According to the 2006 criteria of the AES, PCOS was diagnosed for patients with oligo-anovulation and/or polycystic ovaries (PCO) and clinical and/or biochemical signs of hyperandrogenism (HA) [17]. We ruled out all secondary etiologies, including hyperprolactinemia, thyroid dysfunction, Cushing’s syndrome, congenital adrenal hyperplasia, and virilizing tumors, in all patients using appropriate tests. Pregnant women or those of willingness for pregnancy, patients with contraindications of OC therapy, and those using medications related to PCOS such as hormonal, insulin sensitizers, or antiandrogens drugs within the previous 3 months were excluded from the study.

### Intervention

All participants were alternatively treated for two 6-month treatment periods with OCs. This study had 6 treatment arms with different sequences. All participants were randomly assigned to one of the following treatment groups: Group 1: First treated with Ethinyl estradiol (EE) 30 μg + LNG 0.15 mg and then received the second treatment with OCs containing EE 30 μg + DSG 150 μg. Group 2: First treated with EE 30 μg + LNG 0.15 mg and then received the second treatment with OCs containing EE 35 μg + CPA 2 mg. Group 3: First treated with OCs containing (EE) 30 μg + LNG 0.15 mg and then received the second treatment with OCs containing EE 30 μg + DRSP 3 mg. Group 4: First treated with EE 30 μg + DSG 150 μg and then received the second treatment as EE 30 μg + LNG 0.15 mg. Group 5: First treated with EE 35 μg + CPA 2 mg and then received the second treatment with EE 30 μg + LNG 0.15 mg. Group 6: First treated with EE 30 μg + DRSP 3 mg and then received the second treatment with EE 30 μg + LNG 0.15 mg.

Accordingly, all participants received EE 30 μg + LNG 0.15 mg as the standard treatment. A washout period of 6–8 weeks was considered between the two treatment periods for the elimination of carry-over effects of treatment. Interventions were performed by a trained midwife with the assistance of another individual, who was aware of the type of intervention.

### Randomization and blinding

A blocking or stratification random allocation (block size = 6), using a computer-based random number generator was applied to assign participants to treatment groups. The randomization sequence was prepared before the trial, initiated by an independent statistician. For those patients meeting inclusion criteria and having given written informed consent, the next randomization sequence was assigned by the research assistant according to the schedule. Both the clinical examiner and data analyst were blinded to participants during the trial.

### Outcome measures

For each participant, outcome measures, including clinical, biochemical (androgenic and metabolic), and QOL were collected at 6 time points, i.e. first baseline, at the end of third and sixth months of the first treatment period, after the washout period (second baseline), and at the end of the third and sixth months of the second treatment period, respectively.

Clinical assessments of participants were conducted by only one person who was blinded to groups to minimize any assessor effects. We evaluated all patients for regularity of menstrual cycles; oligomenorrhea was defined as vaginal bleeding episodes at intervals ≥35 days. Menstrual cycle intervals less than 22 days were defined as polymenorrhea. Patients who had no menstrual bleeding for 6 months or longer were considered as amenorrhea [18–20]. Clinical hyperandrogenism was defined by the presence of hirsutism, acne, or androgenic alopecia. The standardized scoring system of m-FG score was used for determining the density of terminal hair at 9 different body sites, i.e., upper lip, chin, chest, upper back, lower back, upper abdomen, lower abdomen, arm, and thigh; a total score ≥ 8 was considered as hirsutism [21, 22]. Acne was diagnosed based on the grading system on the basis of the number of lesions and their spread on the face, back, and chest, and was classified to mild, moderate, moderate to severe, and severe [23]. The Ludwig classification system was used to diagnose androgenic alopecia in patients [24].

Biochemical measurements were performed by an expert laboratory technician under the supervision of a specialist in laboratory sciences. At baseline and follow-ups, fasting (at least 9 h) blood samples were collected between days 3 and 5 of the spontaneous menstrual cycle or progesterone-induced menstrual bleeding. All sera were stored at − 80 °C until the time of testing. Follicle stimulating hormonetotal (FSH) and luteinizing Hormone (LH) were measured by Immunoradiometric assay (IRMA). Androgenic profiels, including testosterone (TT), and DHEAS, were measured by the enzyme immunoassay (EIA), (DRG Diagnostics, GmbH, Germany); SHBG was measured by immune enzymometric assay (IEMA), (Diagnostic biochem Canada Co. Ontario, Canada). FAI was calculated using the formula [TT (nmol/L) × 100/SHBG (nmol/L)] [25]. Metabolic parameters including fasting blood sugar (FBS), TG, TC, LDL cholesterol and HDL cholesterol were measured by colorimetric enzymatic assay (Pars Azmun Co. Tehran, Iran). Insulin was measured by ECLIA (Roche Diagnostics GmbH, Mannheim, Germany). HOMA-IR was calculated by the following formula: [glucose (nmol/L) × insulin (μU/mL)/22.5]. We considered IR as HOMA-IR ≥ 2.63.

We perfomed sonography only at the beginning of the study; it was performed on the same day as the blood samples were collected. Endometrial thickness, ovarian volume, number, diameter, and distribution of the follicles were recorded. The ovaries were considered polycystic when observed as having an ovarian size more than 10 ml) and/or at least 12 follicular cysts measuring 2–9 mm [26].

According to the World Health Organization (WHO) report, QOL is defined as “the individual’s perception of their position in life in the context of the culture and value systems in which they live and in relation to their goals” [27]. In this study, we assessed QOL using a specific and valid health-related quality of life questionnaire for PCOS, developed by Iran, which is consisted of 43 items in six domains, including psychosocial–emotional, self-image, fertility, sexual function, hirsutism, and obesity– menstrual disorders. Items were scored based on the 5-point Likert scale (always, often, sometimes, rarely, never) [28].

### Statistical analysis

In this study, we applied the non-inferiority assumption and intention-to-treat the principle to compare the intervention treatments. EE 30 μg + LNG 0.15 mg was considered as a reference group.

Continuous and categorical variables at the baseline are reported as median (IQR) and percentage, respectively. Effect of oral contraceptives containing EE + DSG, EE + CPA, and EE + DRSP was compared to EE + LNG for the outcomes of interests using the generalized estimating equations (GEE) which are appropriate for longitudinal data. Regression models were adjusted for period, sequence and baseline measure. Confidence intervals (CIs) for effect sizes were estimated through the robust variance estimators obtained on the assumption that the correlation structure was exchangeable. Carryover effect for this 2 × 2 (two sequence-two period) cross over analysis were tested via the first-order-interaction effect of period and treatment, and in case of significant results, the independent working correlation structure was considered to omit intra subject variability [29–31]. Statistical analysis was performed using STATA software (version 13; STATA, INC., College Station, TX, USA). A significant level was considered < 0.05.

### Sample size

To show that the OCs containing LNG are clinically as effective as those containing antiandrogenic progestins including DSG, CPA, and DRSP, CPA, we used a noninferiority hypothesis, where μT is the mean of the test drug, μS is the mean of standard therapy, and δ is a difference of clinical importance.

By rejecting the null hypothesis, we conclude that the difference between the test drug and the standard therapy is less than a clinically meaningful difference (ie, δ), and therefore the test drug is as effective as the standard therapy [32].

We considered 80% power, 0.05 type I error, and ϴ = 0.52, where ϴ is defined as the difference of mean of test drug and standard therapy minus difference of clinical importance divided by standard division. Sample size was calculated from the table introduced by sample size calculations in clinical research [33]. We estimated 25 samples were needed for each group, with 150 total samples needed.

## Results

**Fig. 1:**
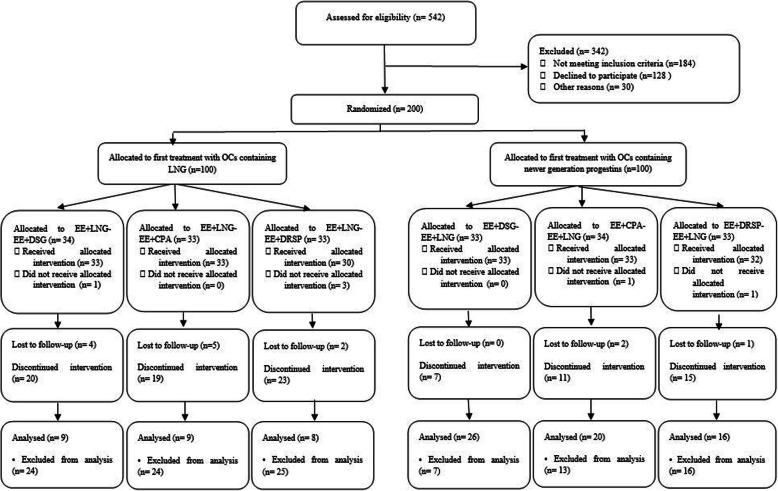
Flow diagram of the study

**Fig. 2:**
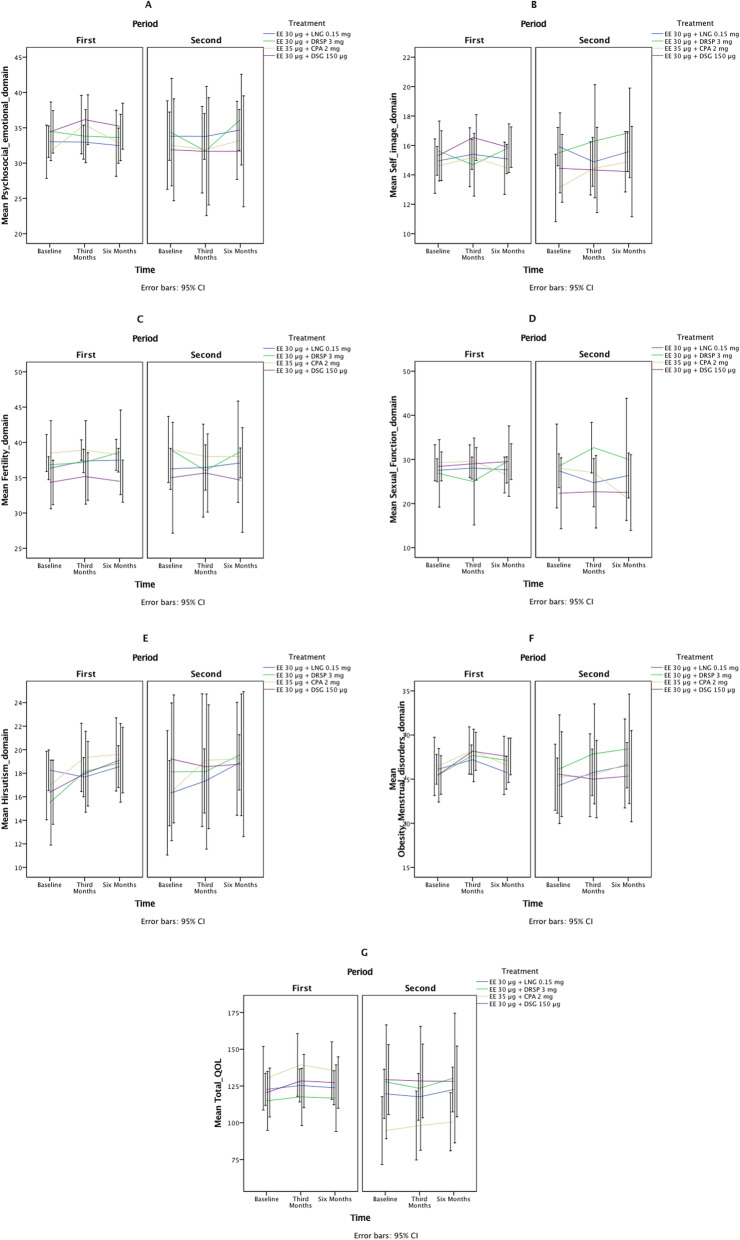
**a**, **b**, **c**, **d**, **e**, **f**, **g** Graphical display of QOL and its domains based on the median and IQR in different timelines and periods (**a**: psychosocial–emotional domain; **b**: Self-image; **c**: fertility domain; **d**: sexual function; **e**: hirsutism; **f**: obesity– menstrual disorders; **g**: total QOL)

Figure 1 presents the flow diagram of this study. Of 200 participants recruited in this study, 14 patients were lost to follow-up and 95 patients discontinued intervention. Finally, after excluding lost to follow-up cases and those who discontinued intervention, data of a total of 88 women with PCOS were analyzed (Group 1: *n* = 9; Group 2: *n* = 9; Group 3: *n* = 8; Group 4: *n* = 16; Group 5: *n* = 20, and group 6: *n* = 26). Table 1 presents the baseline characteristics of patients with PCOS based on the treatment groups. Figure 2a-g depict the total and domains scores of QOL for study groups at baseline. There were no significant differences between treatment groups at baseline variables. No significant differences were observed between study groups at baselines for outcomes (Table 1, Fig. 2a-g).

**Table 1:** Baseline characteristics of patients with PCOS based on the treatment groups

Baseline characteristics	EE 30 μg + LNG 0.15 mg (*n* = 26)	EE 30 μg + DSG 150 μg (*n* = 26)	EE 35 μg + CPA 2 mg (***n*** = 20)	EE 30 μg + DRSP 3 mg (***n*** = 16)	Total	^**€**^***P***-value
**Age, years**	28 (24–33)	28 (23–34)	30 (27–36)	32 (24–37)	29 (25–34)	0.125
**BMI, kg/m2**	25 (23–27)	24 (21–29)	25 (23–28)	26 (22–30)	25 (22–28)	0.251
**Education**						
Diploma and less	0 (0)	2 (7.7)	1 (5.0)	2 (12.5)	5 (5.7)	0.085
Upper diploma	26 (100)	24 (92.3)	19 (95.0)	14 (87.5)	83 (94.3)	
**Marital status**						
Never married	14 (53.8)	14 (53.8)	8 (40.0)	10 (62.5)	46 (52.3)	0.471
Ever married	12 (46.2)	12 (46.2)	12 (60.0)	6 (37.5)	42 (47.7)	
**Parity**						
Nulliparous	3 (25.0)	4 (33.3)	0 (0)	4 (57.1)	11 (26.2)	0.854
Primiparous	6 (50.0)	4 (33.3)	7 (63.6)	2 (28.6)	19 (45.2)	
Multiparous	3 (25.0)	4 (33.3)	4 (36.4)	1 (14.3)	12 (28.6)	
**Job**						
Student	6 (23.1)	5 (19.2)	3 (15.0)	2 (12.5)	16 (18.2)	0.521
Employed	11 (42.3)	10 (38.5)	8 (40.0)	3 (18.8)	32 (36.4)	
Housewife	9 (34.6)	11 (42.3)	9 (45.0)	11 (68.8)	40 (45.5)	
**Infertility status**						
**yes**	3 (11.5)	3 (11.5)	2 (10.0)	1 (6.3)	9 (10.2)	0.147
**no**	23 (88.5)	23 (88.5)	18 (90.0)	15 (93.8)	79 (89.8)	
**Duration of infertility, years**	8 (4–36)	5 (4–18)	24 (24–24)	36 (36–36)	18 (5–24)	
**Menstrual disorder**						
yes	24 (92.3)	26 (100)	20 (100)	16 (100)	86 (97.7)	0.652
no	2 (7.7)	0 (0)	0 (0)	0 (0)	2 (2.3)	
**Duration of Menstrual disorder, months**	120 (60–162)	102 (36–156)	147 (72–204)	157 (42–180)	120 (48–180)	0.124
**Duration of PCOS diagnosis, months**	36 (22–96)	36 (1–54)	30 (12–60)	60 (24–108)	36 (12–87)	0.321
**FSH**	4 (4–5)	5 (4–6)	5 (4–6)	4 (4–5)	5 (4–6)	0.146
**LH**	4 (2–8)	4 (3–6)	4 (3–6)	3 (2–4)	4 (3–6)	0.858
**Total testosterone, ng/ml**	1 (0–1)	1 (1–1)	1 (1–1)	0 (0–1)	1 (0–1)	
**SHBG (nmo/l)**	32 (16–48)	53 (34–95)	53 (34–95)	50 (26–68)	44 (27–67)	0.069
**FAI**	6 (3–10)	4 (2–8)	4 (2–8)	4 (1–6)	4 (2–8)	0.598
**DHEAS**	183 (111–229)	138 (94–216)	138 (94–216)	138 (73–178)	153 (94–206)	0.075
**FBS, mg/dL**	86 (76–91)	86 (78–92)	86 (78–92)	89 (80–98)	86 (78–93)	0.152
**Fasting Insulin**	8 (6–14)	7 (6–13)	7 (6–13)	9 (6–15)	9 (6–13)	0.085
**HOMA-IR**	2 (1–3)	1 (1–3)	1 (1–3)	2 (1–3)	2 (1–3)	0.895
**TG, mg/dL**	76 (60–104)	95 (60–142)	95 (60–142)	89 (80–120)	82 (69–122)	0.064
**TC, mg/dL**	176 (141–199)	170 (147–202)	170 (147–202)	162 (147–174)	169 (146–196)	0.098
**LDL, mg/dL**	99 (77–114)	93 (83–115)	93 (83–115)	91 (80–100)	92 (78–112)	0.581
**HDL, mg/dL**	48 (41–52)	47 (39–56)	47 (39–56)	43 (38–50)	46 (39–52)	0.236

Values are presented as number (%) and median (25–75 interquartile range) for categorical and continuous variables, respectively

Abbreviation: *EE* Ethinylestradiol, *DSG* desogestrel, *CPA* cyproterone acetate, *DRSP* drospirenone, *LNG* levonorgestrel, *BMI* body mass index, *FSH* Follicle stimulating hormone, *LH* Luteinizing Hormone, *SHBG* sex hormone-binding globulin, *DHEAS* Dehydroepiandrosterone sulfate, *FAI* free-androgen index, *TT* total testosterone, *HOMA-IR* homeostasis model assessment of insulin resistance, *TC* total cholesterol, *TG* triglyceride, *LDL* low-density lipoprotein, *HDL* high-density density lipoprotein

^**€**^ *P*-value obtained from chi-squred and kruskal-Wallis tests

Regression models via GEE adjusted for baseline values showed that use of OCs containing DSG, CPA, and DRSP for 3 months was not associated with a significant difference in the total scores of QOL compared to those OCs containing LNG, whereas, after 6 months of treatment, patients treated with OCs containing CPA had more decrease in their total scores of QOL, in comparison to OCs containing LNG (*P* < 0.042) (Tables 2, 3, Fig. 2a-g).

**Table 2:** Estimation of treatment, period, sequence and carryover effects through Generalized Estimating Equation Model (GEE) for quality of life (QOL) at 3 months of therapy

Treatment ^**&**^	Psychosocial–emotional domain	Self-image domain	Fertility domain	Sexual Function domain	Hirsutism domain	Obesity–Menstrual disorders domain	Total QOL
	β ^**&**^(95% CI) ***P***-value	β ^**&**^ (95% CI) ***P***-value	β ^**&**^ (95% CI) ***p***-value	β ^**&**^ (95% CI) ***p***-value	β ^**&**^ (95% CI) ***p***-value	β ^**&**^ (95% CI) ***p***-value	β ^**&**^ (95% CI) ***p***-value
**EE 30 μg + DSG 150 μg (*n*** **= 26)**	0.39 (−12.71, 13.48) 0.954	0.62 (−0.60, 1.83) 0.319	−3.12 (− 15.17, 8.94) 0.612	10.09 (−8.85, 29.04) 0.296	−5.20 (−5.14, −5.14) 0.324	5.96 (−5.16, 17.10) 0.294	20.65 (− 16.74, 58.02) 0.279
**EE 35** μg **+ CPA 2 mg (*n*** **= 20)**	1.66 (−7.71, 11.03) 0.729	0.21 (− 1.07, 1.48) 0.749	−0.94 (−9.67, 7.79) 0.833	10.44 (− 3.29, 24.17) 0.136	5.56 (− 1.82, 12.93) 0.140	5.17 (− 2.78, 13.12) 0.203	20.72 (−5.93, 47.38) 0.128
**EE 30** μg **+ DRSP 3 mg (*n*** **= 16)**	− 1.95 (− 7.66, 3.77) 0.504	− 0.44 (− 1.82, 0.94) 0.534	−2.35 (− 7.42, 2.73) 0.365	3.23 (− 4.84, 11.3) 0.433	2.32 (− 2.10, 6.75) 0.304	2.28 (− 2.53, 7.09) 0.353	3.18 (− 12.63, 18.99) 0.694
**EE 30** μg **+ LNG 0.15 mg (*n*** **= 26)**	Reference	Reference	Reference	Reference	Reference	Reference	Reference
**Sequence**							
EE/LNG as Second Treatment	1.47 (− 2.21, 5.15) 0.433	0.62 (− 0.33, 1.56) 0.203	1.02 (− 3.02, 5.07) 0.620	− 1.28 (− 7.18, 4.63)	− 0.95 (4.39, 2.50) 0.590	0.50 (− 2.82, 3.82) 0.767	5.04 (− 13.63, 23.70) 0.597
EE/LNG as First Treatment	Reference	Reference	Reference	Reference	Reference	Reference	Reference
**Period**							
Second Six Month	−1.90 (− 8.29, 4.50) 0.560	0.10 (− 0.85, 1.04) 0.844	− 1.07 (− 7.07, 4.93) 0.727	5.99 (− 3.45, 15.42) 0.214	2.67 (− 2.34, 7.68) 0.297	2.45 (−2.96, 7.87) 0.374	6.55 (− 11.53, 24.63)
First Six Month	Reference	Reference	Reference	Reference	Reference	Reference	Reference
**Carry over effect**	0.05 (−2.97, 3.07) 0.976	0.34(0.06, 0.45) 0.039*	0.67 (−2.06, 3.40) 0.629	−2.15 (− 6.43, 2.14) 0.326	−1.13 (− 3.52, 1.26) 0.353	−1.33 (− 3.90, 1.24) 0.310	−4.33 (− 12.95, 4.28)

Abbreviation: *EE* Ethinylestradiol, *DSG* desogestrel, *CPA* cyproterone acetate, *DRSP* drospirenone, *LNG* levonorgestrel, *QOL* quality of life

^&^ Beta regression coefficient, (95% CI) and *P*-value showed mean difference estimated via GEE adjusted by baseline values with linear link function and exchangeable working correlation matrix

^$$^ working correlation considered independent in case of significant carryover effect

*P-values < 0.05 are considered significant

**Table 3:** Estimation of treatment, period, sequence and carryover effects through Generalized Estimating Equation Model (GEE) for quality of life (QOL) at 6 months of therapy

Treatment ^**&**^	Psychosocial–emotional domain	Self-image domain	Fertility domain	Sexual Function domain	Hirsutism domain	Obesity–Menstrual disorders domain	Total QOL
	β ^**&**^(95% CI) ***P***-value	β ^**&**^ (95% CI) ***P***-value	β ^**&**^ (95% CI) ***p***-value	β ^**&**^ (95% CI) ***p***-value	β ^**&**^ (95% CI) ***p***-value	β ^**&**^ (95% CI) ***p***-value	β ^**&**^ (95% CI) ***p***-value
**EE 30 μg + DSG 150** μg **(*n*** **= 26)**	9.20 (− 2.48, 20.89) 0.123	4.90 (−1.03, 10.83) 0.105	− 2.13 (− 13.65, 9.39) 0.717	3.73 (− 10.61, 18.07) 0.610	4.57 (−4.25, 13.39) 0.310	10.13 (− 1.03, 21.28) 0.075	33.72 (− 1.19, 68.64)
**EE 35 μg + CPA 2 mg (*n*** **= 20)**	6.43 (− 1.91, 14.77) 0.131	3.10 (−1.13, 7.34) 0.151	0.50 (− 7.86, 8.85) 0.908	2.09 (− 8.31, 12.49) 0.694	4.28 (− 2.01, 10.57) 0.182	7.36 (− 0.61, 15.32) 0.070	25.88 (0.99, 50.77) **0.042** ^*^
**EE 30 μg + DRSP 3 mg (*n*** **= 16)**	3.26 (− 1.75, 8.26) 0.203	2.46 (− 0.09, 5.01) 0.058	− 0.07 (− 4.87, 4.73) 0.976	0.37 (− 5.56, 6.30) 0.903	1.65 (− 2.10, 5.41) 0.389	3.82 (− 0.98, 8.62) 0.119	11.37 (− 3.39, 26.12) 0.131
**EE 30 μg + LNG 0.15 mg (*n*** **= 26)**	Reference	Reference	Reference	Reference	Reference	Reference	Reference
**Sequence**							
EE/LNG as Second Treatment	−3.03 (−6.82, 0.75) 0.116	−1.40 (− 3.26, 0.46) 0.140	−0.11 (− 4.06, 3.84) 0.957	0.47 (− 4.88, 5.83) 0.863	−1.03 (− 4.28, 2.22) 0.536	− 2.35 (− 5.84, 1.15) 0.188	−4.45 (− 23.27, 14.37) 0.643
EE/LNG as First Treatment	Reference	Reference	Reference	Reference	Reference	Reference	Reference
**Period**							
Second Six Month	2.81 (−2.85, 8.48) 0.331	2.02 (− 0.86, 4.90) 0.170	0.08 (− 5.67, 5.83) 0.978	1.26 (− 5.90, 8.41) 0.731	2.21 (− 2.06, 6.48) 0.310	3.91 (− 1.51, 9.33) 0.157	12.16 (−4.72, 29.04) 0.158
First Six Month	Reference	Reference	Reference	Reference	Reference	Reference	Reference
**Carry over effect**	−1.97 (−4.67, 0.73) 0.152	− 1.12 (−2.49, 0.25) 0.110	0.11 (−2.49, 2.72) 0.932	−0.80 (− 4.04, 2.44) 0.628	−1.08 (− 3.12, 0.95) 0.297	−2.14 (− 4.71, 0.44) 0.104	−7.46 (− 15.51, 0.59) 0.069

Abbreviation: *EE* Ethinylestradiol, *DSG* desogestrel, *CPA* cyproterone acetate, *DRSP* drospirenone, *LNG* levonorgestrel, *QOL* quality of life

^&^ Beta regression coefficient, (95% CI) and *P*-value showed mean difference estimated via GEE adjusted by baseline values with linear link function and exchangeable working correlation matrix

^$$^ working correlation considered independent in case of significant carryover effect

*P-values < 0.05 are considered significant

Analysis of domains of QOL showed no significant differences in none of the domains, including Psychosocial–emotional, self-image, fertility, sexual function, hirsutism, and obesity-menstrual disorders after 3–6 months of treatment with DSG, CPA, or DRSP, compared to LNG (Tables 2, 3, Fig. 2a-b).

The sequence effect was not significant in any of the analyses at 3 and 6 months of treatment, indicating that when EE/LNG was considered as the second treatment, QOL did not change, compared to when this compound is the first treatment. We also found no significant period effects for the outcomes at the end of 3 and 6 months of treatment, indicating that these outcomes did not significantly change in the second sixth month of treatment, compared to the first sixth months, regardless of the type of treatment. The carry-over effect was not significant for most outcomes assessed, except for self-image domain at the end of 3 months of therapy (Tables 2 and 3).

## Discussion

This crossover study was carried out to assess the QOL of PCOS patients treated with oral contraceptives based on their progestin component. We found that, in general, OCs with newer progestin such as DSG, CPA, and DRSP had no superiority on increasing scores of QOL different domains, in comparison with older compounds, although usage of products containing CPA was significantly associated with more improvement in total QOL of PCOS patients, compared to those containing LNG after 6th month of treatment.

Quality of life in women with PCOS is multifactorial and has been attributed to clinical, hormonal features, cardio-metabolic abnormalities, in particular obesity, delayed diagnosis, fear about future health, and inadequate information about this syndrome [34]. According to the recent International Evidence-based Guidelines for the Assessment and Management of PCOS, all women with PCOS should be screened and monitored for QOL status to prevent, identify, and manage their health concerns. While these guidelines recommended that improving the QOL of the patients should consider as an important goal of treatment [11, 35, 36], the effect of OCs on psychological health and QOL of PCOS has been investigated only in a limited number of previous studies [12–15]. Available data showed that treatment with OC was associated with a significant improvement in the QOL in a 16-week randomized controlled trial (RCT) [13], a 12-month RCT [12], and a 6-month observational trial [14]. However, it should be kept in mind that none of these studies have been assessed the impact of OCs on the QOL based on their progestin component. Chung et al. [15] during a randomized crossover study showed that the use of medroxyprogesterone acetate and OCs containing CPA for 4 months was not associated with any significant difference in the QOL domains compared to baseline. They suggested that although OCs containing CPA may be a better option for reducing the severity of hyperandrogenism symptoms, there was no difference between these two treatments on the QOL [15]; however, this trial had a study population of adolescents and assessed QOL by SF 36 questionnaire, a nonspecific instrument for PCOS patients. Besides, this study used Rotterdam criteria for the diagnosis of PCOS; therefore study population could have mild phenotypes of disease. A review of the literature suggests that OCs containing an androgenic progestin, such as CPA can reduce not only clinical findings of hyperandrogenism but also the negative impact of these symptoms on the QOL and mental health [37]. In accordance with previous evidence, our results showed that OCs containing DSG, CPA, and DRSP for 3 months had no advantage on the total scores of QOL, compared to those OCs containing LNG, whereas at the end of follow-up (6th month of treatment), patients treated with OCs containing CPA had more improvements in their total scores of QOL, in comparison to OCs containing LNG.

The effects of OCs are attributed to both their estrogenic and progesterone components; Ethinyl estradiol (E2), contained in the pill increases the SHBG level, resulting in the decrease of free androgen levels. Antiandrogenic progestins can block peripheral androgen receptors at target organs, and the reduction of ovarian androgen production. Although all OCs can increase the QOL through the suppression of gonadotropins that can lead to an improvement in clinical manifestations women with PCOS, there is evidence demonstrating that contraceptives with anti-androgen progestins have certain mechanisms, in addition to the main mechanisms to improve HA [38, 39]. CPA is a progestogen known with antiandrogenic properties, which is still commonly prescribed in some countries as a medical treatment, especially for PCOS patients [37]. A recent meta-analysis comparing the effects of OCs with newer progestin showed that although all OCs studies have similar effects on the hormonal profiles of PCOS patients, products containing CPA had more effective to control hyperandrogenism findings of PCOS, findings suggesting positive effects of these antiandrogenic products on QOL of patients [40]. In addition, previous findings of our crossover study revealed the same effects of OCs on clinical findings of hyperandrogenism (HA), whereas products containing LNG were less effective [41].

Previous studies have been suggested that the management of PCOS can improve both physical and psycho-emotional aspects of QOL [10, 12, 13]. To assess the effects of OCs on QOL domains, we used a specific questionnaire with a multi-dimensional concept, which was developed for examining the impact of PCOS or its treatment on psychosocial–emotional, self-image, fertility, sexual function, hirsutism, and obesity-menstrual disorders.

Similar to other chronic diseases, PCOS is associated with an important psychological burden throughout the life of women [7]. Clinical manifestations of androgens excess may influence the feminine identity reflecting on the psycho-emotional status of women, leading to impaired QOL [7]. One cross-sectional study showed a higher prevalence of depression among PCOS patients. They observed that PCOS was managed in only 74.6% of these patients with depression. In addition, poor QOL was observed in 87.8% of these patients, whereas only 9.2% of PCOS patients who received treatment had poor QOL [6], findings suggesting the need for managing psychological disorders in women with PCOS. According to the American Association of Clinical Endocrinologists, the American College of Endocrinology, and the Androgen Excess and PCOS Society recommendations for evaluation and treatment of PCOS patients, all women with PCOS should be screened for anxiety and depressive disorders during the initial examination and its use at further stages of treatment and after it [42]. One large prospective study evaluated the effect of using OCs for 6 months on emotional distress, anxiety, and depression in PCOS patients and showed that the level of depression remained unchanged [14], whereas one RCT showed significant improvements in the mental domain of QOL after 12 months of OC therapy with and without metformin treatment [12]. However, these two studies have been assessed the effects of OCs, regardless of progestin type. A randomized crossover study showed no significant effects of medroxyprogesterone acetate and OCs containing CPA for 4 months on the emotional and mental health of adolescent girls with PCOS compared to their baseline status; this study also showed no difference between these two treatments on psycho-emotional aspects of QOL [15].

Infertility is one of the most common issues among patients with PCOS. Some studies reported a significantly higher rate of depression among PCOS patients with infertility, compared with fertile women with PCOS [9, 43]. We found that the use of OCs containing DSG, CPA, and DRSP was associated with more decrease in the scores of the fertility domain, compared to those with LNG, finding which was non-significant. However, it should be kept in mind that, in Iran, OCs containing LNG commonly prescribe as a contraception method, whereas products containing newer progestins were more well-known as a treatment for hyperandrogenism symptoms. Hence, it seems that patients treated with newer products were more concerned about the adverse effects of these drugs on their reproduction.

There are complexities in the sexual aspect and its related factors in patients with PCOS. Although the negative impact of clinical features of hyperandrogenism on sexual function is well-documented, the role of androgens on sexual function is still debated [7]. On the other hand, there is evidence demonstrating a significant positive relationship between androgen levels and the sexual domain of QoL in PCOS patients [44]. While targeted interventions may help to improve their quality of life through improving sexual relationships, it is unknown whether the treatment of PCOS patients with hyperandrogenism can improve their sexual relationships [7]. In addition, there is no evidence demonstrating the superiority of new generation products in decreasing sexual dysfunction in these patients. This study showed that compared to patients treated with OCs containing LNG, those treated with OCs containing newer progestin (DSG, CPA, and DRSP) had similar effects on sexual function of patients.

Hirsutism is another common problem with a considerable impact on the QOL. Previous studies suggest the role of hirsutism in developing anxiety and mood disorders [10]. One cross-sectional showed that 87% of PCOS patients with hirsutism had poor QOL; they found that those patients with hirsutism had lower QOL than non-hirsute patients. In this study, most patients had not been used any treatment for hirsutism [6]. Two studies showed a significant improvement of the hirsutism aspect after 6 months [14] and 12 months [12] of OC therapy; however, these studies did not compare different OCs regarding their progestin component. Although it seems reasonable that compounds with an anti-androgenic component can enhance the QOL by more improvement of hirsutism, our study showed the non-inferiority of these OCs, compared to the older products on this domain of QOL.

While there is a big controversy regarding the effects of oral contraceptives on adiposity indices, it is well documented that all oral contraceptives, regardless of their progestin type, can improve menstrual regularity, mainly through suppress hypothalamus pituitary gonadal [45] and lead to increased QOL [14]. This study showed similar effects on OCs on the physical domain of QOL (obesity-menstrual disorders). Sidra et al. [6] showed that obese patients with PCOS had lower QOL scores. On the other hand, weight loss interventions can associate with an improvement in QOL these patients, although mostly these interventions are unsuccessful, mainly due to the apparent inability of patients with PCOS to lose weight [6]. Altinok et al. [12] observed significant improvements in body mass index (BMI) domain of QOL after medical treatment with metformin, but not with OC therapy, indicating that lifestyle interventions may be a better therapeutic option in weight loss compared to hormonal agents. Cinar et al. reported an improvement of menstrual regularity aspect of QOL after 6 months of using OCs [14]. In agreement with literature, we found that there was any significant difference in menstrual regularity based on OC type, indicating that all OCs can be effective for improving menstrual disorders.

In the crossover study, a washout period of 6 to 8 weeks was considered between two periods of treatment to eliminate carryover effects. Since the treatment effects could be influenced by the order in which treatments may be received; hence this study was designed with 6 treatment arms based on the different treatment sequences. We did not significantly observe significant sequence and carryover effects for almost all outcomes, demonstrating that washout duration was sufficient. As expected, the most common side effects of OCs were headache, dizziness, nausea, and spotting; these complaints declined at the end of the 6th month of treatment.

The strengths of this study include its crossover design, long duration of follow-up, comparing OCs based on their progestin component, and using specific health-related QOL for PCOS patients. This study also has several limitations that should be considered. As most crossover studies, and despite all of our approaches, there was a more loss to follow-up than initially expected; however, it should be kept in mind that patients left the study mostly because of unwillingness to continue treatment for non-medical reasons and they had no difference in their baseline characteristics, such as age and BMI. Although the loss to follow-ups extremely reduced the sample size, each study group had 7 or more participants; recent guidelines for calculating sample size in cross-over trials recommended having more than at least 5 participants in each study group [46]. GEE analysis can also partially overcome missing data [47]. It should be also considered that the study population was diagnosed based on the AES criteria, which detect sever PCOS phenotypes; hence, our results may be not generalizable for mild phenotypes diagnosed using Rotterdam criteria.

## Conclusions

This crossover study demonstrated non-inferiority of OCs with newer generation progestins such as DSG, CPA, and DRSP on different domains of QOL, in comparison with older compounds, although usage of products containing CPA was significantly associated with more improvement in total QOL of PCOS patients, compared to those containing LNG after 6th month of use.

## Data Availability

Data is Available after publication if requested, via email to corresponding author.

## Abbreviations

PCOS: Polycystic ovary syndrome
QOL: Quality of life
OCs: Oral contraceptives
LNG: Levonorgestrel
DSG: Desogestrel
CPA: Cyproterone acetate
DRSP: Drospirenone
CONSORT: Consolidated Standards of Reporting Trials
AES: Androgen Excess Society
RIES: Research Institute for Endocrine Sciences
EE: Ethinyl estradiol
WHO: World Health Organization
GEE: Generalized estimating equations
CI: Confidence intervals
RCT: Randomized controlled trial
BMI: Body mass index.
